# Maternal fruit and vegetable or vitamin C consumption during pregnancy is associated with fetal growth and infant growth up to 6 months: results from the Korean Mothers and Children’s Environmental Health (MOCEH) cohort study

**DOI:** 10.1186/s12937-018-0410-6

**Published:** 2018-11-12

**Authors:** Won Jang, Hyesook Kim, Bo-Eun Lee, Namsoo Chang

**Affiliations:** 1 Department of Nutritional Science and Food Management, Ewha Womans University, Seoul, South Korea; 2 Environmental Health Research Division, National Institute of Environmental Research, Incheon, South Korea

**Keywords:** Fruits, Vegetables, Vitamin C, Birth weight, Birth length, Fetal growth, Infant growth

## Abstract

**Background:**

Based on data obtained from pregnant women who participated in the Mothers and Children’s Environmental Health (MOCEH) study in South Korea, we aimed to determine whether maternal intake of fruits and vegetables or vitamin C is associated with fetal and infant growth.

**Methods:**

A total of 1138 Korean pregnant women at 12–28 weeks gestation with their infants were recruited as study participants for the MOCEH. Intake of fruits and vegetables or vitamin C during pregnancy was assessed by a 1-day 24-h recall method. Fetal biometry was determined by ultrasonography at late pregnancy. Infant weight and length were measured at birth and 6 months.

**Results:**

A multiple regression analysis after adjusting for covariates showed that maternal intake of fruits and vegetables was positively associated with the biparietal diameter of the fetus and infant’s weight from birth to 6 months. Also, maternal vitamin C intake was positively associated with the abdominal circumference of the fetus and infant birth length. In addition, there was a significant inverse relationship between consumption of fruits and vegetables (below the median compared to above the median of ≥519 g/d) and the risk of low growth (<25th percentile) of biparietal diameter (odds ratio (OR): 2.220; 95% confidence interval (CI): 1.153–4.274) and birth weight (OR: 1.434; 95% CI: 1.001–2.056). A significant inverse relationship also existed between vitamin C consumption (below vs above the estimated average requirement (EAR) of ≥85 mg/d) and the risk of low growth (<25th percentile) of birth weight (OR: 1.470; 95% CI: 1.011–2.139), weight from birth to 6 months (OR: 1.520; 95% CI: 1.066–2.165), and length at birth (OR: 1.579; 95% CI: 1.104–2.258).

**Conclusions:**

An increased intake of fruits and vegetables or vitamin C at mid-pregnancy is associated with increased fetal growth and infant growth up to 6 months of age.

## Background

Vitamin C, also known as ascorbic acid, acts as an antioxidant by scavenging reactive oxygen and nitrogen species in an aqueous environment [1] and plays a major role in defense against increased oxidative stress during pregnancy [2]. The human body cannot produce or adequately store vitamin C and, consequently, it must regularly be obtained through the diet. Vitamin C levels decrease during pregnancy, due to inadequate intake [3] and physiological changes in pregnancy, leading to hemodilution [4, 5].

The increased consumption of fruit and vegetables, an excellent source of vitamin C, during pregnancy has been shown to be positively associated with birth weight [6, 7]. In contrast, inadequate dietary intake of vitamin C may result in adverse outcomes, such as low birth weight (below 5% standard birth weight for gestational age) [8] and preterm delivery (birth that occurs before the 37th week of pregnancy) [9]. Two prospective cohort studies, one in the UK [10] and the other in India [6], reported a positive association between maternal vitamin C intake during pregnancy and birth weight.

To the best of our knowledge, only two studies have examined the association between maternal vitamin C status and birth outcomes in South Korea. One of these prospective cohort studies (*n* = 239) [11] indicated that maternal serum vitamin C levels during the second trimester were positively correlated with birth weight and length in full-term babies. In the other survey (*n* = 383) [12], a high maternal concentration of vitamin C was significantly linked to increased infant weight and head circumference during the first 3 years of life. However, both these studies involved a small sample size and neither determined growth measurements (such as fetal biometry) before the baby was born. These two studies focused mostly on the relationship between vitamin C status during pregnancy, especially blood concentration of vitamin C and infant growth.

The easiest way to raise the blood levels of vitamin C is to consume vitamin C through fruits and vegetables. A previous observation identified dietary vitamin C intake as the variable with the greatest influence on plasma vitamin C concentrations [13]. Measuring vitamin C concentrations in the body has more advantages than dietary intake, but dietary intake data represent significant measures of habitual vitamin C consumption. Therefore, it is also necessary to consider the relevance of infant growth based on the intake of fruits and vegetables, and vitamin C, regarding the practical application. Many studies [6–10] have reported an association between the consumption of either fruits and vegetables or vitamin C with infant growth, but there have been no in-depth studies of Korean women in this regard. Therefore, this research investigated whether maternal intake of vitamin C, based on a diet abundant in fruit and vegetables, is associated with fetal and infant growth, namely birth length and weight, in pregnant Korean women.

## Methods

### Study subjects and data collection

**Fig. 1:**
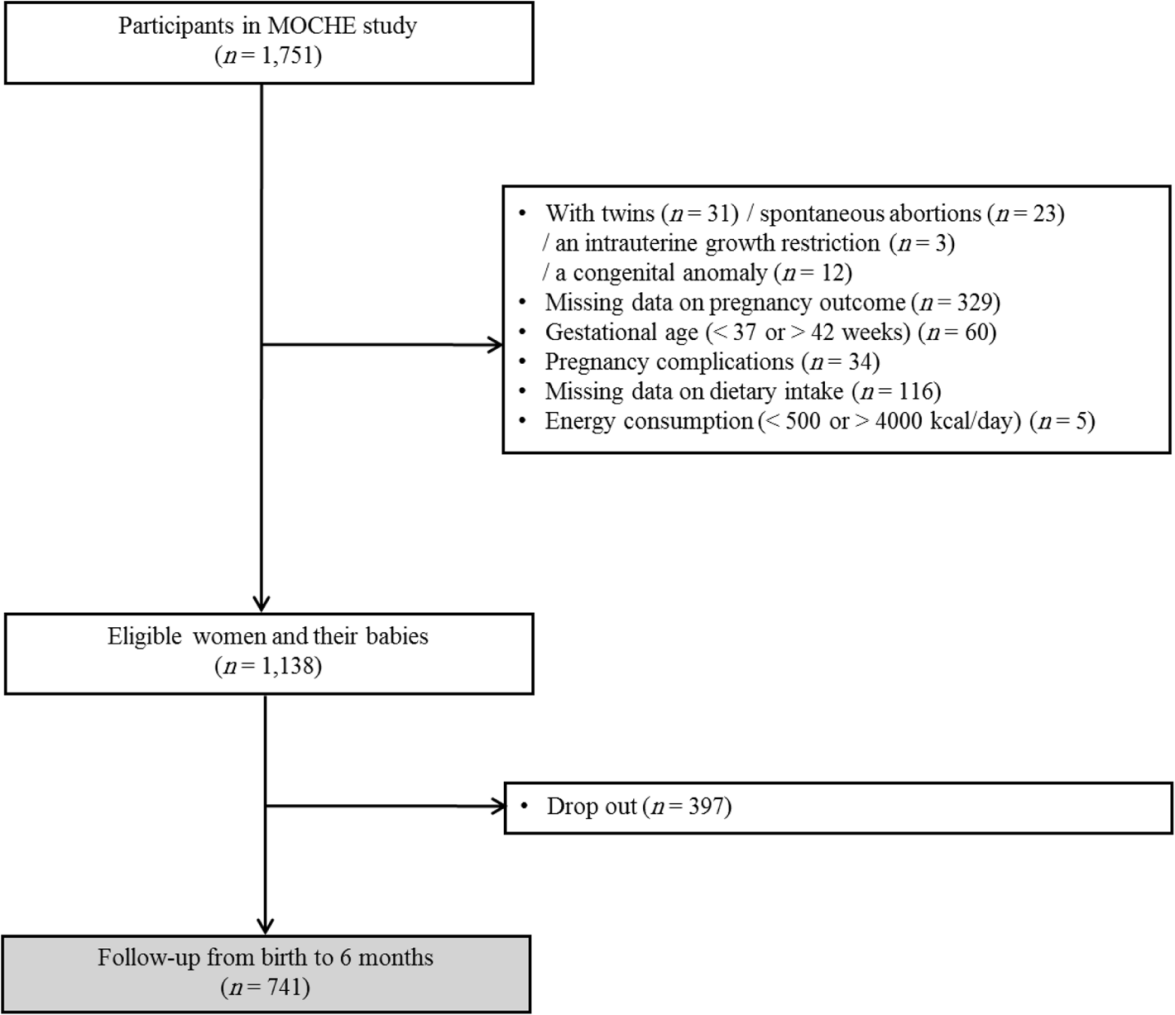
Flow chart of the subject inclusion and exclusion criteria in the study

This study was based on data obtained by the Mothers and Children’s Environmental Health study (MOCEH), which is an ongoing multi-center (Seoul, metropolitan city; Ulsan, industrial region; and Cheonan, medium-sized urban area), prospective cohort study in South Korea. Detailed information about the MOCEH study has been reported elsewhere [14]. The study protocol was attentively reviewed and authorized by the Institutional Review Boards at Ewha Womans University School of Medicine, Dankook University Hospital, and Ulsan University Hospital, respectively, and the informed consent for participation was acquired from all subjects. The study subject selection process is outlined in Fig. 1. The MOCEH study population consists of 1751 pregnant women at 12–28 weeks of gestation and their infants after birth. The subjects were recruited between August 2006 and December 2010. Of these 1751 subjects, 31 women carrying twins, 23 who suffered spontaneous abortions, 3 who had an intrauterine growth restriction, and 12 whose fetus had a congenital anomaly were excluded. Of the remaining 1682 subjects, 329 whose pregnancy outcome data were not available due to drop out from the study, 60 whose pregnancy lasted less than 37 or more than 42 weeks, and 34 who had pregnancy complications (hypertension or/and diabetes) were excluded. From the 1259 subjects left, 116 whose dietary intake data were not collected, and 5 whose total energy consumption was < 500 kcal/d or > 4000 kcal/d were also excluded. Thus, 1138 women and their babies were finally included in the analysis. Among these subjects, babies with data related to fetal biometric measurements, such as biparietal diameter (*n* = 703), abdominal circumference (*n* = 589) and femur length (*n* = 689) at late pregnancy (between 29 and 42 weeks gestation) were analyzed. We were only able to follow-up 741 infants from birth to 6 months (Fig. 1).

### Dietary assessment

Maternal dietary intake data during mid-pregnancy were collected for one 24-h recall for intake on the day before the participant’s visit to the center. The 24-h dietary recall was administered by experienced, well-trained dietary interviewers, who instructed the respondents to recall and describe all foods and beverages, including nutritional supplements, they had consumed over the past 24 h. The dietary information was processed using a computerized nutrient intake assessment program (CAN-Pro 4.0, Korean Nutrition Society, Seoul, Korea).

### General characteristics and pregnancy outcomes

The method used to survey the general characteristics of the subjects has been described in detail elsewhere [15]. Information on demographic and socioeconomic status and health-related behaviors [maternal age (years), height (cm), and weight (kg) at pre-pregnancy; education status; family income; parity; supplement use; smoking; alcohol consumption] were gathered by well-trained interviewers. Urine samples were collected and stored frozen until they could be delivered to a specialized laboratory for analysis. During pregnancy, maternal oxidative stress status was assessed by measuring the urinary levels of malondialdehyde (MDA; an oxidation product of lipoperoxidation), which is indicated to be a useful biomarker of oxidative stress in pregnant women [16]. The urinary MDA levels were analyzed by high-performance liquid chromatography (HPLC) with fluorescence detection. The urinary level of cotinine (a reliable biomarker of nicotine exposure) was measured by HPLC-isotope dilution tandem mass spectrometry (Agilent 6410 Triple Quad LCMS, Agilent Technologies, Santa Clara, CA, USA). The data were adjusted relative to the urinary creatinine level, to correct for urine volume. As indices for fetal biometry, the biparietal diameter, abdominal circumference, and femur length were assessed by ultrasonography at late pregnancy, by a certified ultrasonographer at Ewha Womans University Hospital. The gestational age, determined by ultrasound measurement, was obtained from medical records. Neonatal gender, gestational age at delivery (weeks), birth weight (kg), and birth length (cm) data were also retrieved from medical records. Gestational age at delivery was inferred by subjects’ self-reports of the last menstrual period and was also estimated via ultrasonography. Weight (±0.1 kg) and length (±0.1 cm) at 6 months were measured using a DS-B02 infantometer (Dong Sahn Jenix Co. Ltd., Seoul, Korea), by laying each infant in the center of the scale. When participants visited the hospital after birth, mothers were asked to complete questionnaires that pertained to information about breastfeeding status, as well as any change in demographic characteristics, family disease history, and environmental conditions, since the previous visit.

### Statistical analysis

A descriptive analysis was conducted to present general characteristics, health-related behaviors, dietary intakes, and growth measurements, including pregnancy outcomes, of the study population. Continuous variables were expressed as mean ± standard deviation (SD), and the categorical variables were indicated as frequency and percentage. The food and nutrient intake data, and urinary cotinine levels were log-transformed to normalize their distributions. A multiple regression analysis was conducted to investigate the relationship between maternal fruit and vegetable/vitamin C intake and fetal growth. For estimating the difference in fetal growth, according to maternal fruit and vegetable/vitamin C intake level, the subjects were divided into two groups [fruits and vegetables, below/above the median value of 519 g/d] [vitamin C, below/above the estimated average requirement (EAR) of 85 mg/d] and the data then analyzed by a generalized linear model test, after controlling for confounding factors. The adjusted odds ratios (ORs) (with 95% confidence interval, CI) for the prevalence of low growth (<25th percentile) in infants of mothers with low fruit and vegetable/vitamin C intake levels were calculated by logistic regression analysis. Variables that were considered to be confounding factors (having the potential to influence the relationship between maternal vitamin C intake and weight and length at birth, observed from the univariate analysis of these data or reported from other literature) were included as covariates. These covariates included maternal age (years), pre-pregnancy BMI (kg/m^2^), log-transformed urinary cotinine level (μg/g creatinine), neonatal gender (male or female), gestational age at delivery (weeks), and the use of supplements (yes or no), residential area (metropolitan area, industrial area, urban area), parity (0 or ≥ 1), father’s height (cm), and log-transformed intakes of energy (kcal), vitamin E (mg alpha-tocopherol equivalents, α-TE), and β-carotene (μg). The infant’s growth at 6 months was additionally adjusted for breastfeeding status (exclusive breastfeeding or formula-feeding or combined) at 6 months. For repeated measures analyses from birth to 6 months, mixed models (for continuous dependent variables) and general estimating equation models (for categorical dependent variables) were used. SAS 9.3 (SAS Institute, Cary, NC, USA) was used for all data analyses, and the level of significance was set at *P* < 0.05.

## Results

### Characteristics of the study population

The participants had a mean age of 30.2 ± 3.6 years and a pre-pregnancy BMI of 21.3 ± 3.1 kg/m^2^. Approximately 55.7% of the subjects took dietary supplements (Table 1).

**Table 1:** Characteristics of the pregnant women and infants

	*n*	Mean ± SD or *n(%)*
General characteristics		
Age, years	1136	30.2 ± 3.6
Pre-pregnancy BMI*,* kg/m^2^	1099	21.3 ± 3.1
Education	1078	
≤ High school		293 (27.2)
≤ University		714 (66.2)
≥ Graduate school		71 (6.7)
Family monthly income, USD	1064	
< 2000		288 (27.1)
2000–4000		596 (53.5)
≥ 4000		207 (19.5)
Parity	967	
0		485 (50.2)
≥ 1		482 (49.8)
Supplement user	1138	634 (55.7)
Urinary cotinine, μg/g creatinine	1057	20.9 ± 159.2
Urinary MDA, μmol/g creatinine	1036	1.8 ± 1.4
Dietary intakes		
Total food intake, g/d	1138	1368.0 ± 450.0
Fruit and vegetables, g*/*d	1138	594.4 ± 365.4
Energy, kcal/d	1138	1774.6 ± 502.6
Carbohydrates, g/d	1138	276.4 ± 82.0
Protein, g/d	1138	68.7 ± 25.0
Fat, g/d	1138	47.0 ± 24.2
Vitamin C, mg/d	1138	134.8 ± 99.2
Vitamin E, mg α-TE/d	1138	17.1 ± 8.5
β-carotene, μg/d	1138	2703.5 ± 2275.6
Fetal biometry at late pregnancy		
Biparietal diameter, cm	703	8.94 ± 0.56
Abdominal circumference, cm	589	32.13 ± 2.54
Femur length, cm	689	6.85 ± 0.53
Pregnancy outcomes		
Neonatal gender (male)	1132	582 (51.4)
Gestational age at delivery, weeks	1138	39.1 ± 1.1
Birth weight, kg	1138	3.3 ± 0.4
Birth length, cm	1138	50.5 ± 2.5
Breastfeeding status, yes	741	480 (64.8)
Infants characteristics at 6 months		
Weight, kg	741	10.1 ± 1.0
Length, cm	740	76.7 ± 2.5

*BMI* Body mass index, *MDA* Malondialdehyde, *α-TE* Alpha-tocopherol equivalents

The average vitamin C intake of the pregnant women was 134.8 ± 99.2 mg/d, and 36.1% did not meet the EAR (85 mg/d) for the Korean Dietary Reference Intake. The average fruit and vegetable consumption of the pregnant women was 594.4 ± 365.4 g/d. Gestational age at the time of ultrasound measurement, fetal biparietal diameter, abdominal circumference, and femur length were 36.4 ± 2.5 weeks, and 8.9 ± 0.1, 32.1 ± 2.5, and 6.9 ± 0.5 cm, respectively. Gestational age at delivery was 39.1 ± 1.1 weeks, birth weight was 3.3 ± 0.4 kg, and birth length was 50.5 ± 2.5 cm. The general characteristics did not differ significantly between the included and excluded subjects (data not shown).

### Correlation between fruit and vegetable/vitamin C intake and urinary MDA levels during pregnancy

According to the correlation analysis (Table 2), maternal intake of fruits and vegetables during pregnancy was positively correlated (*r* = 0.727, *P* < 0.0001) with vitamin C intake. Also, maternal fruit and vegetable (*P* < 0.05) and vitamin C (*P* < 0.05) intakes were both negatively correlated with urinary MDA levels at mid-pregnancy, respectively.

**Table 2:** Coefficients of correlation (*r*) between fruit and vegetable intake, vitamin C intake and urinary malonaldehyde (MDA) concentrations at mid-pregnancy (*n* = 1036)

Intake	Urinary MDA concentration	Fruit and vegetable intake	Vitamin C intake
Fruit and vegetables	−0.064*	1	0.727**
Vitamin C	−0.065*	0.727**	1

Intake data were log-transformed to conform to normal distribution. *p < 0.05. **p < 0.0001

### Association of maternal fruit and vegetable/vitamin C intake with fetal growth at late pregnancy

Multiple regression analysis indicated that maternal fruit and vegetable intake during pregnancy was not associated with the biparietal diameter (β = 0.045, *P* = 0.0511) at late pregnancy. A general linear model adjusted for covariates revealed that biparietal diameter was higher in fetuses whose mothers had a fruit and vegetable consumption above the median value (≥519 g/d) than in fetuses of mothers with a lower fruit and vegetable intake (*p* = 0.0245). Similar analyses for vitamin C intake during pregnancy showed a significant result in the abdominal circumference. Maternal vitamin C intake was positively associated with abdominal circumference (β = 0.279, *P* = 0.0354) at late pregnancy. Abdominal circumference in fetuses of mothers with intakes of vitamin C above the EAR (≥85 mg/d) was significantly higher compared to fetuses of mothers with intakes below the EAR (*P* = 0.0234). After controlling for confounding factors in the multiple logistic regression analysis, a significant inverse relationship was noted between fruit and vegetable consumption and the risk of low biparietal diameter (<25th percentile). Compared with fetuses whose mothers had a fruit and vegetable consumption above the median value (≥519 g/d), those of mothers with a lower fruit and vegetable intake had a higher risk (OR: 2.220; 95% CI: 1.153–4.274) of low biparietal diameter (<25th percentile) at late pregnancy (Table 3).

**Table 3:** Association of maternal fruit and vegetable/vitamin C intake with fetal growth at late pregnancy

Intake	At late pregnancy								
	Biparietal diameter (cm)			Abdominal circumference (cm)			Femur length (cm)		
	*n*	β (SE)/ lsmean ± SD	p	*n*	β (SE)/ lsmean ± SD	p	*n*	β (SE)/ lsmean ± SD	p
Fruit and vegetables									
Continues									
Unadjusted	703	0.004 (0.031)	0.8919	589	0·046 (0.161)	0.7743	689	0.032 (0.030)	0.2835
Adjusted^a^	515	0.045 (0.023)	0.0511	433	0·175 (0.135)	0.1964	507	0.001 (0.023)	0.9747
Categorical^a^									
< median (519 g/d)		8.92 ± 0.02	0.0245		32.11 ± 0.12	0.2464		6.88 ± 0.02	0.8144
≥ median (519 g/d)		8.99 ± 0.02			32.30 ± 0.12			6.87 ± 0.02	
Vitamin C									
Continues									
Unadjusted	703	0.006 (0.030)	0.8457	589	0.045 (0.155)	0.7730	689	−0.022 (0.029)	0.4344
Adjusted^b^	515	0.030 (0.023)	0.1898	433	0.279 (0.132)	0.0354	507	0.021 (0.023)	0.3463
Categorical^b^									
< EAR (85 mg/d)		8.94 ± 0.03	0.3342		31.94 ± 0.14	0.0234		6.86 ± 0.02	0.3632
≥ EAR (85 mg/d)		8.97 ± 0.02			32.35 ± 0.11			6.89 ± 0.02	
ORs (95% CI) for low fetal biometry (<25th percentile)									
Fruit and vegetables									
< median (519 g/d)	2.220 (1.153–4.274)		0.0170	1.695 (0.900–3.192)		0.1023	0.772 (0.388–1.537)		0.4613
≥ median (519 g/d)	1 (ref)			1 (ref)			1 (ref)		
Vitamin C									
< EAR (85 mg/d)	1.468 (0.753–2.865)		0.2600	1.682 (0.848–3.335)		0.1367	1.139 (0.535–2.423)		0.7359
≥ EAR (85 mg/d)	1 (ref)			1 (ref)			1 (ref)		

β value is the coefficient from the multiple regression analysis

Least squares means (lsmeans) is a mean estimated from a general linear model

EAR is estimated average requirement

ORs is odds ratios

Intake data were log-transformed for normal distribution

^a^Adjusted for maternal age, pre-pregnancy body mass index (BMI), urinary cotinine level (log-transformed), infant gender, gestational age at the time of ultrasound measurement, use of supplements, residential area, parity, father’s height, energy intake (log-transformed)

^b^Adjusted for maternal age, pre-pregnancy BMI, urinary cotinine level (log-transformed), infant gender, gestational age at the time of ultrasound measurement, use of supplements, residential area, parity, father’s height, energy intake (log-transformed), vitamin E intake (log-transformed), β-carotene intake (log-transformed)

### Association of maternal fruit and vegetable/vitamin C intake with infant growth at birth and up to 6 months

Fetal biometric measurements, such as biparietal diameter, abdominal circumference, and femur length, were positively correlated (*P* < 0.0001) with infant birth weight and length (data not shown). Based on the multiple regression analysis (Table 4), maternal fruit and vegetable intake during pregnancy was not associated with infant weight and length at birth and 6 months, respectively. However, when we conducted mixed models analysis for repeated measures from birth to 6 months, fruit and vegetable intake was positively associated with infants’ weight (*β* = 0.054, *P* = 0.0184). Birth weight in neonates born to mothers who consumed above the median fruit and vegetable value (≥519 g/d) was significantly higher than those born to mothers who consumed below the median value (*P* = 0.0024); these results were also observed in mixed models analysis for birth to 6 months (*P* = 0.0077). Maternal vitamin C intake was positively associated with infant birth length (*β* = 0.314, *P* = 0.0056). Birth weight (*P* = 0.0400) and length (*P* = 0.0010) in neonates born to mothers who consumed above the EAR (≥85 mg/d) of vitamin C were significantly higher than in neonates born to mothers who consumed below the EAR (*P* = 0.0010). After adjusting for covariates, a significant negative relationship was observed between maternal fruit and vegetable consumption and low infant weight at birth (<25th percentile) [OR (95% CI) for below the median compared to above the median (≥519 g/d): 1.434 (1.001–2.256)]. There was also a significant negative correlation between the vitamin C consumption and low weight at birth (<25th percentile) [OR (95% CI) for below the EAR compared to above the EAR (≥85 mg/d): 1.470 (1.011–2.139)]. A similar trend was revealed between vitamin C intake and low weight from birth to 6 months (<25th percentile) [OR (95% CI) for below the EAR compared to above the EAR (≥85 mg/d): 1.520 (1.066–2.165)] and low length at birth (<25th percentile) [OR (95% CI) for below the EAR compared to above the EAR (≥85 mg/d): 1.579 (1.104–2.258)].

**Table 4:** Association of maternal fruit and vegetable/vitamin C intake with infants’ weight and length from birth to 6 months

Intake	Weight (kg)									Length (cm)								
	At birth			At 6 months			Birth to 6 months			At birth			At 6 months			Birth to 6 months		
	*n*	β (SE)/lsmean ± SD	p	*n*	β (SE)/lsmean ± SD	p	*n*	β (SE)/lsmean ± SD	p	*n*	β (SE)/lsmean ± SD	p	*n*	β (SE)/lsmean ± SD	p	*n*	β (SE)/lsmean ± SD	p
Fruit and vegetables																		
Continues																		
Unadjusted	1138	0.014 (0.017)	0.4295	741	−0.058 (0.058)	0.3150	1138	0.013 (0.017)	0.4609	1138	0.116 (0.101)	0.2471	740	0.073 (0.162)	0.6520	1138	0.120 (0.101)	0.2329
Adjusted^a^	816	0.031 (0.020)	0.1182	559	−0.056 (0.064)	0.3848	639	0.054 (0.023)	0.0184	816	0.172 (0.119)	0.1479	558	−0.043 (0.189)	0.8214	639	0.170 (0.133)	0.2032
Categorical^a^																		
< median (519 g/d)		3.22 ± 0.03	0.0024		10.09 ± 0.11	0.6718		3.23 ± 0.04	0.0077		50.44 ± 0.20	0.1620		76.82 ± 0.34	0.5126		50.78 ± 0.22	0.2993
≥ median (519 g/d)		3.29 ± 0.03			10.12 ± 0.11			3.31 ± 0.04			50.65 ± 0.20			76.98 ± 0.32			50.95 ± 0.22	
Vitamin C																		
Continues																		
Unadjusted	1138	0.015 (0.016)	0.3537	741	0.018 (0.053)	0.7344	1138	0.014 (0.016)	0.3941	1138	0.197 (0.092)	0.0333	740	0.231 (0.148)	0.1182	1138	0.202 (0.092)	0.0294
Adjusted^b^	816	0.035 (0.019)	0.0677	559	0.017 (0.060)	0.7803	639	0.037 (0.021)	0.0808	816	0.314 (0.113)	0.0056	558	0.054 (0.178)	0.7624	639	0.289 (0.125)	0.0214
Categorical^b^																		
< EAR (85 mg/d)		3.22 ± 0.04	0.0400		10.07 ± 0.12	0.4290		3.23 ± 0.04	0.0565		50.20 ± 0.21	0.0010		77.00 ± 0.36	0.7231		50.53 ± 0.24	0.0068
≥ EAR (85 mg/d)		3.28 ± 0.03			10.14 ± 0.11			3.29 ± 0.04			50.74 ± 0.19			76.90 ± 0.31			51.02 ± 0.21	
ORs (95% CI) for low birth outcomes and low growth up to 6 months (<25th percentile)																		
Fruit and vegetables																		
< median (519 g/d)	1.434 (1.001–2.056)		0.0494	0.863 (0.627–1.186)	0.3631	1.311 (0.939–1.829)			0.1117	1.316 (0.933–1.857)		0.1178	0.875 (0.636–1.204)		0.4126	1.152 (0.845–1.570)	0.3714	
≥ median (519 g/d)	1 (ref)			1 (ref)		1 (ref)				1 (ref)			1 (ref)			1 (ref)		
Vitamin C																		
< EAR (85 mg/d)	1.470 (1.011–2.139)		0.0439	0.910 (0.645–1.284)	0.5916	1.520 (1.066–2.165)			0.0206	1.579 (1.104–2.258)		0.0124	1.182 (0.837–1.669)		0.3416	1.262 (0.900–1.770)	0.1773	
≥ EAR (85 mg/d)	1 (ref)			1 (ref)		1 (ref)				1 (ref)			1 (ref)			1 (ref)		

β value is the coefficient from the multiple regression analysis

Least squares means (lsmeans) is a mean estimated from a general linear model

EAR is estimated average requirement

ORs is odds ratios

Intake data were log-transformed to follow a normal distribution

For repeated measures analyses from birth to 6 months, mixed models (when the dependent variables are continuous) and general estimating equation models (when dependent variables are categorical) were used

^a^Adjusted for maternal age, pre-pregnancy body mass index (BMI), urinary cotinine level (log-transformed), infant gender, gestational age at delivery, use of supplements, residential area, parity, father’s height, energy intake (log-transformed) at birth; additionally, adjusted for breastfeeding status at 6 months

^b^Adjusted for maternal age, pre-pregnancy BMI, urinary cotinine level (log-transformed), infant gender, gestational age at delivery, use of supplements, residential area, parity, father’s height, energy intake (log-transformed), vitamin E intake (log-transformed), β-carotene intake (log-transformed) at birth; additionally, adjusted for breastfeeding status at 6 months

## Discussion

In the present large cohort study, we investigated the association of maternal fruit and vegetable and vitamin C intakes during mid-pregnancy with increased fetal growth and infant growth up to 6 months of age, in a Korean population. We found maternal fruit and vegetable intake had a positive association with the biparietal diameter of the fetus and infant weight at birth to 6 months. Also, maternal vitamin C intake was positively associated with the abdominal circumference of the fetus and infant birth length.

Similar results, demonstrating fruit and vegetable consumption during pregnancy favorably influences fetal growth, have been reported by previous investigators. Rao et al. [9] and Mikkelsen et al. [7] documented a significant increase in birth weight with increased fruit and vegetable consumption (as a food group). Ramón et al. [17] observed an increased intake of vegetables, but not fruit, during pregnancy was associated with birth length and weight. Loy et al. [18] noted that vegetable intake was linked to birth length and head circumference, and fruit intake was correlated with birth weight and length, and head circumference.

In our study, the multiple logistic regression analysis revealed a significant inverse relationship between fruit and vegetable consumption and the risk of low growth (<25th percentile) of biparietal diameter and weight at birth. In line with this result, there was a significant inverse relationship between consumption of vitamin C (an abundant nutrient in fruit and vegetables) and the risk of low (<25th percentile) weight at birth, weight from birth to 6 months, and length at birth. Prior research has demonstrated that the vitamin C contained in fruit and vegetables may contribute to both, placental functions and optimal immune system functioning [19], which are paramount for fetal development.

Along with maternal vitamin consumption, we investigated maternal oxidative stress (MDA level), considering that it might be increased under normal pregnancy environments and cause adverse birth outcomes. Although the correlation coefficient value is very low, the maternal fruit and vegetable, and vitamin C intakes were negatively correlated with the oxidant marker (MDA) levels at mid-pregnancy, in agreement with the literature [16]. This factor could partly explain the underlying reason for our findings that women consuming low amounts of fruit and vegetables, and vitamin C had low birth outcomes’, with the offspring having low growth up to 6 months. Antioxidant defense systems are vital to protecting tissues and cells from damage caused by oxidative stress. Consequently, an imbalance between increased oxidative stress and decreased antioxidant defense can adversely affect pregnancy outcomes, including fetal growth retardation [20]. Increased vitamin C consumption (supplement or via fruits and vegetables) during pregnancy may be advantageous to birth size due to its role in the endogenous antioxidant defense system. In a previous epidemiological study in Spain of 586 newborns, an increase in dietary vitamin C intake during pregnancy was implicated to reduce the association between estimated maternal dietary benzo[a]pyrene (a cigarette smoke carcinogen) intake and infant size at birth [21]. Also, maternal fruit and vegetable intake may modify the association between birth weight and bulky DNA adduct levels, which are purported to be a sensitive biomarker of genotoxic agents [22].

Another possibility is that vitamin C, an essential cofactor for two key enzymes (lysyl and prolyl hydroxylase) in collagen biosynthesis, is beneficial for cartilage and bone development [23]. Some experimental studies have found that vitamin C inadequacy can result in a decreased proliferation of chondrocytes in the growth plate and impaired matrix synthesis [24] in mice, and low bone mineral density and bone length in a scorbutic guinea pig model [25]. However, further investigation is needed to unveil the true effects of vitamin C intake during pregnancy on fetal bone growth and collagen metabolism.

During normal prenatal development, fetal vitamin C concentrations in plasma are higher than maternal levels. One study demonstrated an approximately two-fold higher vitamin C level in newborns at birth than in their mothers [26], implying that a high vitamin C status is of particular importance in the fetus. However, our subjects did not meet the optimal vitamin C range. The mean vitamin C intake in our subjects was 134.3 mg/d, and about 36.2% and 51.2% of pregnant women had vitamin C intakes below the EAR and recommended nutrient intake, respectively (data not shown). The vitamin C intake status in our pregnant women is higher than that reported in China [27], Iran [28], England [29], New Zealand [30], and Brazil [31], but lower than that documented in Thailand [32] and the USA [33].

The main vitamin C sources were fruits (40.3%) and vegetables (46.5%) (data not shown), but the correlation between fruit and vegetable/vitamin C with fetal and infant growth was partly inconsistent. Notably, in the biparietal diameter, a significant inverse relationship between fruit and vegetable intake and a low growth rate (<25th percentile) was observed, whereas mothers with a relatively lower vitamin C intake did not have a higher risk of low biparietal diameter. It may be that fruits and vegetables have played a role in other ways, such as provide unexpected bioactive components that are not vitamin C. The high number of bioactive components are found in fruits and vegetables, such as vitamins, minerals, phytosterols, phenolic compounds [34], carotenoids, and fiber. Also, fruit and vegetable intakes may be a pivotal part of a healthy lifestyle and dietary pattern and may be associated with other health factors besides the variables we have adjusted [35, 36].

Our study had some limitations. First, we did not measure plasma vitamin C concentrations, which are considered to be a more objective means to assess vitamin C status than dietary vitamin C intake reports alone, due to the limited amounts of samples for other measurements (e.g., environmental heavy metals and toxins). Second, the maternal dietary intake data from a single 24-h recall may not be sufficient to determine typical daily intake, due to a probable and considerable intra-individual variability in food and nutritional intake. However, the well-trained dietitians applied standard protocols to assist the subjects’ recollection of their daily diet and thereby minimize any potential bias. Regarding intra-individual variability of nutrient intake, as part of the 4th Korean National Health and Nutrition Examination Survey in 2009 [37], the intra-individual variation in nutrient intake measured by a single 24-h dietary recall was compared to an original 1-d dietary interview. Regarding total energy and, particularly, vitamin C intake, comparable values were obtained from each interview. Besides, the seasonality of food can be a problem for 24-h dietary recall. To minimize its undesired effect, the season could be added as a covariate in our analysis. However, there was no difference between fruits and vegetables and vitamin C intake according to the season (data not shown). This observation seems to be because the timing of the pregnant women’s recruitment, that is, the period of the dietary survey was evenly distributed. Therefore, we decided that it was not necessary to include the season as a covariate. In addition, a Greek study investigating the impact of seasonality on the mean nutrient intake of children and adolescents reported that the intake of vitamin C and the macronutrient contribution to the total energy intake, estimated by two 24-h recalls of different seasons, were statistically similar between the two periods (spring/summer and autumn/winter) [38]. Evaluation of potential genotypes that can affect oxidative stress metabolism, as well as the enzymatic activity for heterozygotes and assessment of some specific oxidative markers, might also have improved the reliability of our results.

The strengths of our study include the sample size and the research strategy, considering the strictly-controlled prospective birth cohort design of the MOCEH study in Korea. Also, this research collected reliable data from medical records. Furthermore, an adjustment for crucial confounders having the potential to affect the correlation between maternal vitamin C intake and birth outcomes of newborns were considered, in detail, for more precise analysis.

Fetal growth is an important determinant of health and disease throughout a human’s lifetime. Interestingly, a prospective birth cohort study in Brazil noted a positive association of birth length with blood pressure at 11 years of age, whereas birth weight and blood pressure were not associated [39]. It exemplifies the importance of the relationship between maternal vitamin C and infant birth length as modifiers that could affect children later in life.

## Conclusion

In conclusion, an increased intake of dietary vitamin C, which is abundant in fruit and vegetables, at mid-pregnancy is associated with increased fetal growth and infant growth up to 6 months of age.

## Abbreviations

CI: Confidence interval
DRI: Dietary Reference Intake
EAR: Estimated average requirement
HPLC: High-performance liquid chromatography
IUGR: Intrauterine growth restriction
KNHANES: Korean National Health and Nutrition Examination Survey
MDA: Malondialdehyde
MOCEH: Mothers’ and Children’s Environmental Health
OR: Odds ratio
RNI: Recommended nutrient intake
SD: Standard deviation
